# Complete Genome Sequence of a Wild-Type Measles Virus Isolated during a 2016 Winter Outbreak in a Refugee Settlement in Calais, France

**DOI:** 10.1128/genomeA.00009-17

**Published:** 2017-03-09

**Authors:** Julia Dina, Justine Hamel, Denise Antona, Astrid Vabret

**Affiliations:** a CHU de Caen, Department of Virology, Caen, France; b Normandy University, UNICAEN, EA2656 GRAM, Caen, France; c National Reference Center (NRC) for Measles and Paramyxoviridae Respiratory Viruses, Caen, France; d French National Public Health Agency (Santé publique France), Saint-Maurice, France

## Abstract

Measles outbreaks are regularly reported in European countries despite efforts to improve vaccination coverage. In January 2016, an outbreak occurred in a refugee settlement in Calais, France. We report here the complete genome sequence of a wild-type measles virus isolated from a health care worker (MVi/Calais. FRA/01.16) infected during this outbreak.

## GENOME ANNOUNCEMENT

The measles virus (MeV) belongs to the *Paramyxoviridae* family, *Morbilivirus* genus. MeV is responsible for one of the most contagious diseases, measles. Measles infection is preventable by vaccination and MeV circulation can be stopped if the vaccination coverage is superior to 95% (1).

In January 2016, a measles outbreak was reported in northern France, in the Calais refugee settlement. In this region, no cases were reported since September 2013. A total of 13 cases were reported during this outbreak, three of them involving health care workers (2). One of the health care workers was a volunteer in the camp and the two others worked at the Calais hospital where the patients with measles symptoms were addressed for consultation. MeV was isolated from an oropharyngeal swab collected from the nurse who took in charge the first refugee case in the Calais hospital. As she became ill, she was hospitalized in the intensive care unit for pneumonia. Although her vaccination status was initially declared as vaccinated with two doses in the 1990s, she appeared to actually be unvaccinated against measles (confirmed on documents). The MeV involved was detected by a specific reverse transcription (RT)-PCR in the three samples collected from this nurse, the oropharyngeal swab, urines, and blood (3). The Ct of the target in the oropharyngeal sample was 1,947. The sample was diluted and filtrated and 100 µl of the diluted sample was deposited on Vero-hSlam cells. A cytopathogenic effect, a syncytium formation, was observed 5 days later. Total RNA was extracted using the Qiagen EZ1 DSP virus kit 48. A DNAc was obtained using Superscript III Reverse Transcriptase, Invitrogen. For the amplification of the genome, 35 couples of primers were used, 30 of them were originals and the other five were already published (4). The size of the PCR products was between 500 and 1,000 nucleotides. They were sequenced by the Sanger method. The 3′ leader and 5′ trailer nucleotides were obtained using a system of primers who extend the genome with 50 nucleotides on both sides.

In agreement with WHO guidelines, the virus isolate was named MVi/Calais.FRA/01.16[B3] (5). Complete genome analysis revealed that the Calais virus has 15,894 nucleotides and shared 99% identity with the measles strain MVi/Manchester.GBR/31.13 wt, the closest relative measles virus strain. Arrangement genes of MVi/Calais.FRA/01.16 follows the usual 3′-N-P/V/C-M-F-H-L-5′ order and obeys the rule of six.

The WHO recommendations were applied for genotyping. The MeaNS (Measles Nucleotide Surveillance) analysis of the 450 nucleotides encoding the carboxy-terminal region of the N protein classified the strain as a B3 genotype, subtype MVs/Allada.BEN/3.10. The exact match was identified with MVi/Napoli.ITA/45.15 and MVi/Sokoto.NGA/35.15.

The WHO reference sequence closest to MVi/Calais.FRA/01.16 is MVi/Ibadan.NGA/0.97.

### Accession number(s).

The complete genome sequence of the MVi/Calais.FRA/01.16[B3] isolate was deposited in GenBank under the accession number KX838946.

## References

[1] van Boven M, Kretzschmar M, Wallinga J, O’Neill PD, Wichmann O, Hahné S 2010 Estimation of measles vaccine efficacy and critical vaccination coverage in a highly vaccinated population. J R Soc Interface 7:1537–1544. doi:10.1098/rsif.2010.0086. 20392713 PMC2988255

[2] Jones G, Haeghebaert S, Merlin B, Antona D, Simon N, Elmouden M, Battist F, Janssens M, Wyndels K, Chaud P 2016 Measles outbreak in a refugee settlement in Calais, France: January to February 2016. Euro Surveill 21:30167. doi:10.2807/1560-7917.ES.2016.21.11.30167. 27020578

[3] Hübschen JM, Kremer JR, De Landtsheer S, Muller CP 2008 A multiplex Taqman PCR assay for the detection of measles and rubella virus. J Virol Methods 149:246–250. doi:10.1016/j.jviromet.2008.01.032. 18353451

[4] Penedos AR, Myers R, Hadef B, Aladin F, Brown KE 2015 Assessment of the utility of whole genome sequencing of measles virus in the characterisation of outbreaks. PLoS One 10:e0143081. doi:10.1371/journal.pone.0143081. 26569100 PMC4646484

[5] World Health Organization 2012 Measles virus nomenclature update: 2012. Wkly Epidemiol Rec 87:73–81. 22462199

